# HHV8-Positive Castleman Disease and In Situ Mantle Cell Neoplasia within Dermatopathic Lymphadenitis, in Longstanding Psoriasis

**DOI:** 10.3390/diagnostics11071150

**Published:** 2021-06-24

**Authors:** Magda Zanelli, Luca Stingeni, Maurizio Zizzo, Giovanni Martino, Francesca Sanguedolce, Andrea Marra, Barbara Crescenzi, Stefano A. Pileri, Stefano Ascani

**Affiliations:** 1 Pathology Unit, Azienda USL-IRCCS di Reggio Emilia, 42112 Reggio Emilia, Italy; 2 Dermatology Section, Department of Medicine and Surgery, University of Perugia, 06129 Perugia, Italy; luca.stingeni@unipg.it; 3 Surgical Oncology Unit, Azienda USL-IRCCS di Reggio Emilia, 42122 Reggio Emilia, Italy; maurizio.zizzo@ausl.re.it; 4 Clinical and Experimental Medicine PhD Program, University of Modena and Reggio Emilia, 41121 Modena, Italy; 5 Pathology Unit, Azienda Ospedaliera Santa Maria di Terni, University of Perugia, 05100 Terni, Italy; gio.martino@gmail.com (G.M.); s.ascani@aospterni.it (S.A.); 6 Pathology Unit, Policlinico Riuniti, University of Foggia, 71122 Foggia, Italy; francesca.sanguedolce@unifg.it; 7 Centre of Hemato-Oncology Research (CREO), Institute of Hematology, University and Hospital of Perugia, 06129 Perugia, Italy; andrea.marra1987@gmail.com; 8 Laboratory of Molecular Medicine, CREO, Azienda Ospedaliera di Perugia, University of Perugia, 06129 Perugia, Italy; barbara.crescenzi@ospedale.perugia.it; 9 Haematopathology Division, European Institute of Oncology—IEO IRCCS, 20141 Milan, Italy; stefano.pileri@unibo.it

**Keywords:** psoriasis, castleman disease, HHV8, mantle cell, lymphoma, dermatopathic lympadenitis

## Abstract

A 73-year-old man presented with multiple lymphadenopathy. He had a 20-year history of palmoplantar psoriasis evolved to a diffuse erythrodermic picture in the last two years. Topic and systemic medications including prednisolone, acitretin, anti-IL17 (ixekizumab), TNF inhibitor (adalimumab), anti-IL23 (guselkumab), methotrexate, cyclosporine, and phosphodiesterase 4 inhibitor (apremilast) were ineffective. Repeated skin biopsies excluded mycosis fungoides, confirming psoriasis; molecular analysis of T-cell receptor genes ruled out clonality. The axillary lymph node histology documented a dermatopathic lymphadenitis, often associated with chronic cutaneous inflammatory diseases. At an accurate morphological evaluation, features of HHV8-positive multicentric Castleman disease were observed. Moreover, in a few follicles, in situ mantle cell neoplasia was identified. The translocation t(11;14)(q13;q32), characteristic of mantle cell lymphoma, and the monoclonal IGH gene rearrangement were present. HHV8 DNA was identified on plasma sample. Multicentric Castleman disease in psoriatic patients is a rare event and it might be favored by the immunomodulatory treatment in longstanding psoriasis. Multicentric Castleman disease patients are predisposed to developing simultaneous or subsequent lymphoma. In situ mantle cell neoplasia often behaves indolently, although it may progress to overt mantle cell lymphoma. Rituximab achieved a good control of psoriasis. Unfortunately, the patient developed Staphylococcus aureus sepsis for which he is currently on antibiotic therapy.

A 73-year-old man presented with multiple superficial and profound lymphadenopathy. The patient had a 20-year history of palmoplantar psoriasis. In the last two years he developed a diffuse erythrodermic picture (Figure 1).

Topic and systemic medications including prednisolone, acitretin, anti-IL17 (ixekizumab), TNF inhibitor (adalimumab), anti-IL23 (guselkumab), methotrexate, cyclosporine, and phosphodiesterase 4 inhibitor (apremilast) obtained little benefit. Repeated skin biopsies ruled out mycosis fungoides, as the histopathology was consistent with psoriasis and molecular analysis excluded T-cell receptor (TCR) gene clonality.

The excised axillary lymph node showed distinctive reactive features of dermatopathic lymphadenitis with paracortical hyperplasia and melanin-pigmented macrophages (Figure 2), typically seen in chronic cutaneous inflammatory diseases.

Additionally, atrophic, hyalinized germinal centers with prominent penetrating venules (Figure 3) and interfollicular polytypic plasma cells were noted. Scattered Human Herpesvirus 8 (HHV8)-positive cells (Figure 4) within the mantle and interfollicular regions were identified. A subtle rim of cyclin D1-positive mantle cells was disclosed within few reactive-appearing follicles (Figure 5).

These findings were consistent with HHV8-positive multicentric Castleman disease (MCD) of mixed type associated with in situ mantle cell neoplasia (isMCN). The translocation t(11;14)(q13;q32) characteristic of mantle cell lymphoma (MCL) and the monoclonal IGH gene rearrangement were identified by fluorescence in situ hybridization (FISH) analysis and molecular study, respectively. HHV8 DNA (71 copies/mL) was identified by polymerase chain reaction (PCR) on the plasma sample.

The patient received infusions of the monoclonal antibody rituximab (375 mg/m^2^, once every two weeks x four doses). Rituximab is also reported to be helpful in treating MCD [1]. The treatment was completed about a month before our case description, achieving good control of his psoriasis. Unfortunately, the patient developed Staphylococcus aureus sepsis, for which he is currently on antibiotic treatment.

According to the current 2017 World Health Organization (WHO) classification, HHV8-positive MCD belongs to HHV8-associated lymphoproliferative disorders [1,2,3,4,5]. 

MCD commonly occurs in immunosuppressed patients, being often reported in association with HIV infection, but it may also develop in HIV-negative individuals, especially originating from HHV8-endemic regions [1]. MCD is usually negative for Epstein Barr virus, whereas it is strongly associated with Human Herpes virus 8 (HHV8) infection, detected in almost all HIV-positive cases and in 50% of HIV-negative ones [1].

It presents often with constitutional symptoms (e.g., fever, asthenia, weight loss), generalized lymphadenopathy, splenomegaly and hepatomegaly [1]. Laboratory tests reveal cytopenia, hypoalbuminemia, hypergammaglobulinemia, elevated serum levels of inflammatory markers (C-reactive protein and IL-6) [1]. It usually follows an unfavorable outcome [1].

In patients with psoriasis, HHV8-positive MCD is very rarely reported [6]. Its emergence has been supposed to be linked to the immunomodulatory treatment, particularly in severe and longstanding psoriasis [6]. It is worth mentioning the relevance of discussing with the patient about possible risks and benefits of the immunomodulatory treatment, as it was adequately done in the present case.

MCD patients have an increased risk of developing simultaneous or subsequent lymphomas of different subtypes, which generally follow an aggressive course [1,7,8,9].

MCL is a non-Hodgkin B-cell lymphoma, traditionally considered an aggressive disease, although more indolent forms, including isMCN, are currently well known [1].

isMCN represents a rare event, usually identified incidentally. It generally follows an indolent course, although it may rarely progress to overt MCL after many years [1].

isMCN has been exceptionally reported in association with Castleman disease [10].

## Figures and Tables

**Figure 1:**
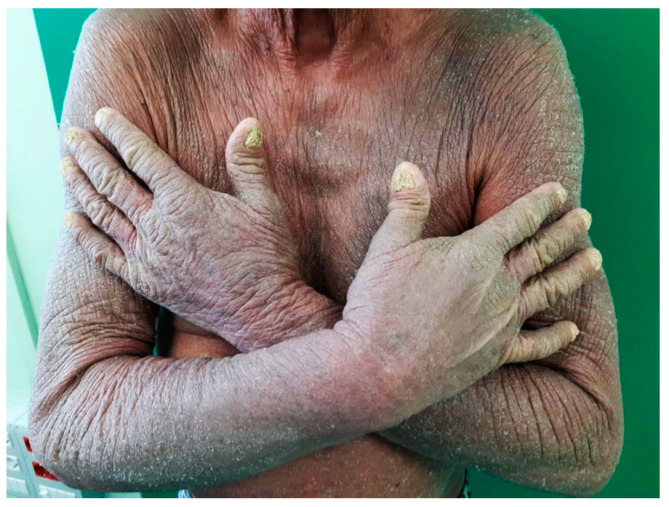
Clinical image showing a diffuse erythrodermic picture of psoriasis.

**Figure 2:**
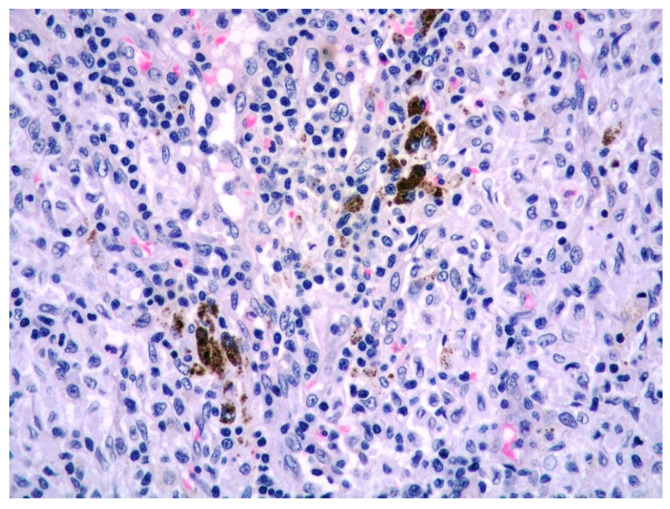
High power view of dermatopathic lymphadenitis with melanin-laden macrophages (HE, 400× magnification).

**Figure 3:**
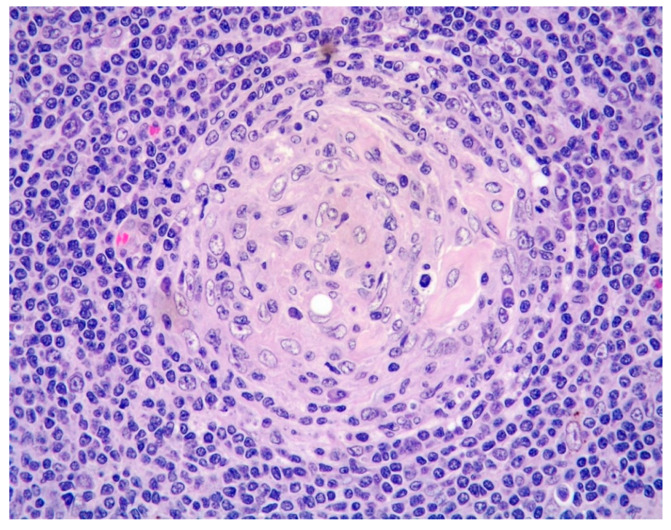
Hyalinized germinal centers with penetrating venules characteristic of Castleman disease (HE, 400× magnification).

**Figure 4:**
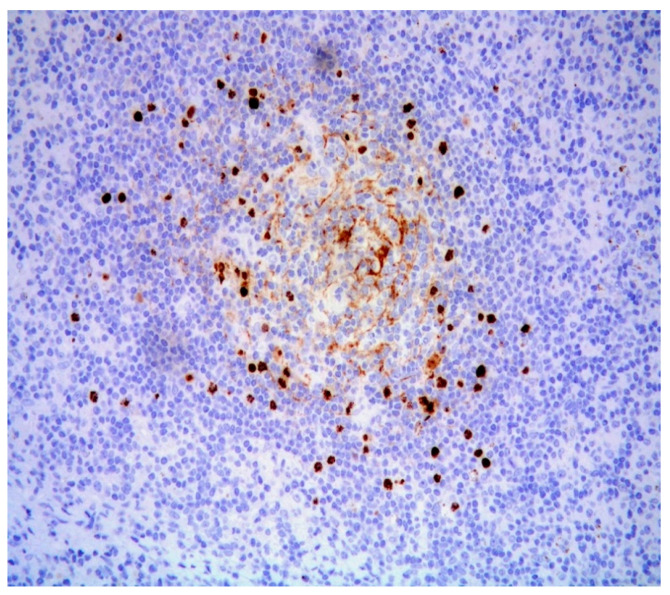
Scattered cells positive for HHV8 immunostaining (magnification 200×).

**Figure 5:**
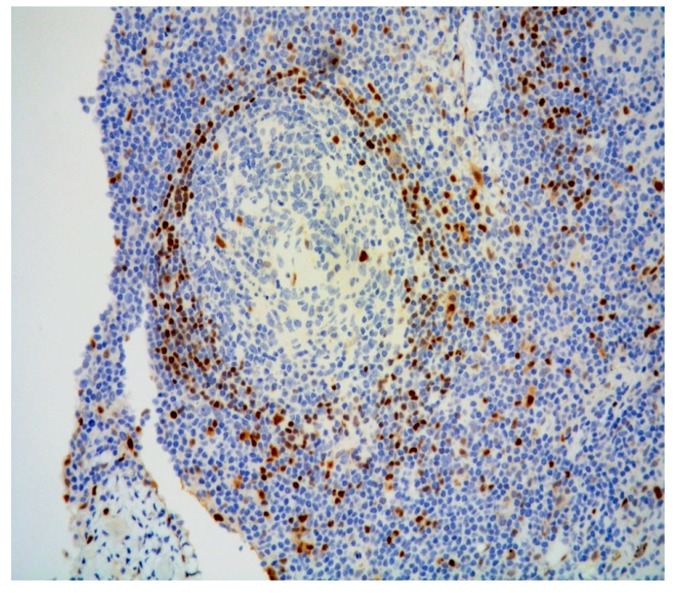
Cyclin D1-positive cells within the mantle zone of a reactive-appearing follicle (immunostain, 200× magnification).

## Data Availability

The data presented in this study are available from the corresponding author upon request.
